# 2-(2-Hy­droxy-3-meth­oxy­phen­yl)-6*H*-perimidin-6-one

**DOI:** 10.1107/S1600536811006465

**Published:** 2011-02-26

**Authors:** Hoong-Kun Fun, Kullapa Chanawanno, Suchada Chantrapromma

**Affiliations:** a X-ray Crystallography Unit, School of Physics, Universiti Sains Malaysia, 11800 USM, Penang, Malaysia; b Crystal Materials Research Unit, Department of Chemistry, Faculty of Science, Prince of Songkla University, Hat-Yai, Songkhla 90112, Thailand

## Abstract

The mol­ecule of the title perimidine derivative, C_18_H_12_N_2_O_3_, is essentially planar, the dihedral angle between the benzene and perimidine rings being 3.25 (5)°. The hy­droxy and meth­oxy groups lie in the plane of the benzene ring to which they are bound [O—C—C—C = 179.96 (11)° and C—O—C—C = −177.96 (12)°]. An intra­molecular O—H⋯N inter­action generates an *S*(6) ring motif. In the crystal, mol­ecules are linked by pairs of C—H⋯O inter­actions into dimers, which generate *S*(16) ring motifs. These dimers are arranged into sheets parallel to the *ac* plane and further stacked down the *b* axis by π–π inter­actions, with centroid–centroid distances in the range 3.5066 (8)–3.7241 (7) Å.

## Related literature

For hydrogen-bond motifs, see: Bernstein *et al.* (1995 ▶). For bond-length data, see: Allen *et al.* (1987 ▶). For background to perimidines and their applications, see: Claramunt *et al.* (1995 ▶); del Valle *et al.* (1997 ▶); Herbert *et al.* (1987 ▶); Llamas-Saiz *et al.* (1995 ▶); Pozharskii & Dalnikovskaya (1981 ▶); Varsha *et al.* (2010 ▶). For related structures, see: Llamas-Saiz *et al.* (1995 ▶); Varsha *et al.* (2010 ▶). For the stability of the temperature controller used in the data collection, see: Cosier & Glazer (1986 ▶).
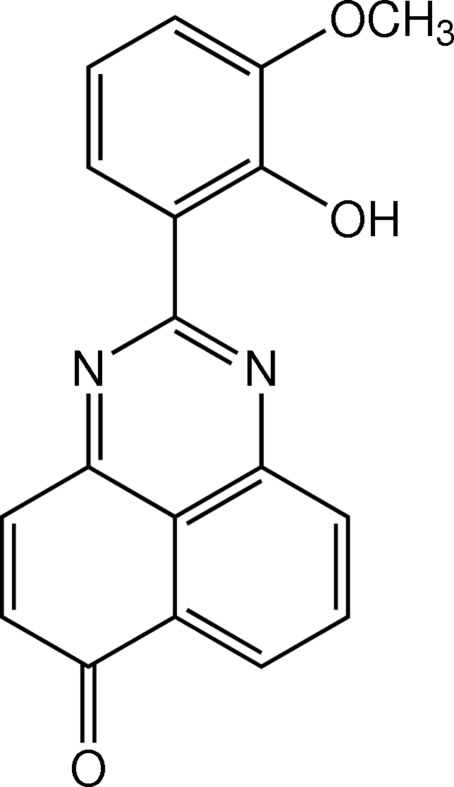


## Experimental

### 

#### Crystal data

C_18_H_12_N_2_O_3_
*M* *_r_* = 304.30
Monoclinic, 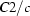

*a* = 25.4718 (17) Å
*b* = 7.0666 (3) Å
*c* = 15.0815 (6) Å
β = 94.373 (3)°
*V* = 2706.8 (2) Å^3^
*Z* = 8
Mo *K*α radiation
μ = 0.10 mm^−1^
*T* = 100 K
0.67 × 0.11 × 0.05 mm

#### Data collection

Bruker APEX DUO CCD area-detector diffractometer
Absorption correction: multi-scan (*SADABS*; Bruker, 2005 ▶) *T*_min_ = 0.933, *T*_max_ = 0.994
42939 measured reflections
6529 independent reflections
3471 reflections with *I* > 2σ(*I*)
*R*_int_ = 0.072

#### Refinement

*R*[*F* ^2^ > 2σ(*F* ^2^)] = 0.066
*wR*(*F* ^2^) = 0.210
*S* = 1.02
6529 reflections
255 parameters
All H-atom parameters refined
Δρ_max_ = 0.57 e Å^−3^
Δρ_min_ = −0.29 e Å^−3^

### 

Data collection: *APEX2* (Bruker, 2005 ▶); cell refinement: *SAINT* (Bruker, 2005 ▶); data reduction: *SAINT*; program(s) used to solve structure: *SHELXTL* (Sheldrick, 2008 ▶); program(s) used to refine structure: *SHELXTL*; molecular graphics: *SHELXTL*; software used to prepare material for publication: *SHELXTL* and *PLATON* (Spek, 2009 ▶).

## Supplementary Material

Crystal structure: contains datablocks global, I. DOI: 10.1107/S1600536811006465/sj5107sup1.cif

Structure factors: contains datablocks I. DOI: 10.1107/S1600536811006465/sj5107Isup2.hkl

Additional supplementary materials:  crystallographic information; 3D view; checkCIF report

## Figures and Tables

**Table 1:** Hydrogen-bond geometry (Å, °)

*D*—H⋯*A*	*D*—H	H⋯*A*	*D*⋯*A*	*D*—H⋯*A*
O2—H1*O*2⋯N2	0.91 (2)	1.73 (2)	2.5583 (15)	151 (2)
C15—H15⋯O2^i^	0.970 (18)	2.437 (18)	3.3686 (17)	160.8 (12)

Symmetry code: (i) 
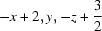
.

**Fig. 1.**
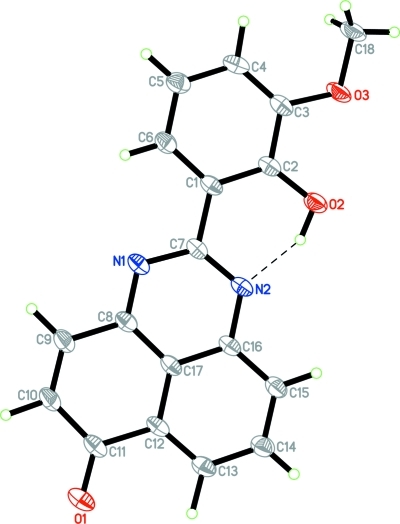
The molecular structure of the title compound, showing 50% probability displacement ellipsoids and the atom-numbering scheme. An intramolecular hydrogen bond is drawn as a dashed line.

**Fig. 2.**
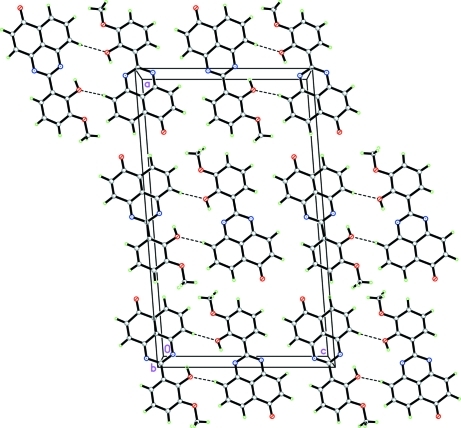
The crystal packing of the title compound viewed down the b axis. Hydrogen bonds were drawn as dashed lines.

## supplementary crystallographic information

### Comment

Perimidines (*peri*-naphtho-fused perimidine ring systems) have received wide interests due to their applications in photophysics (del Valle *et al.*, 1997), usage as coloring materials for polyester fibers (Claramunt *et al.*, 1995) and fluorescent materials (Varsha *et al.*, 2010). They are also noted for their biological activity displaying antiulcer, antifungal, antimicrobial and antitumor properties (Claramunt *et al.*, 1995; Herbert *et al.*, 1987; Pozharskii & Dalnikovskaya, 1981). In an attempt to synthesize a Co(II) Schiff base complex by the reaction of *o*-vanillin, 1,8-diaminonaphthalene and CoCl_2_.6H_2_O, the unexpected product was the perimidine derivative, (I), reported here, Fig 1.

In the molecule of the title perimidine derivative (I), C_18_H_12_N_2_O_3_, the perimidine ring system (N1–N2/C7–C17) is planar with an *r.m.s* deviation 0.0126 (11)Å. The whole molecule is essentially planar with the dihedral angle between the perimidine and phenyl rings being 3.25 (5)°. Both the hydroxy and methoxy groups are co-planar with the attached benzene ring with torsion angles O2–C2–C3–C4 = 179.96 (11)° and C18–O3–C3–C2 = -177.96 (12)°. An intramolecular O—H···N interaction generates an S(6) ring motif (Bernstein *et al.*, 1995) and helps to stabilize the planarity of the molecule (Fig. 1). Bond distances are normal (Allen *et al.*, 1987) and are comparable to those found in related structures (Llamas-Saiz *et al.*, 1995; Varsha *et al.*, 2010).

In the crystal structure (Fig. 2), the molecules are linked by two C—H···O interactions (Table 1) into dimers which generate S(16) ring motifs. These dimers are arranged into sheets parallel to the *ac* plane and further stacked down the *b* axis by π–π interactions with centroid to centroid  distances *Cg*_1_···*Cg*_2_^ii^= 3.7241 (7) Å; *Cg*_2_···*Cg*_3_^ii^= 3.5066 (8) Å; *Cg*_2_···*Cg*_4_^ii^= 3.7055 (8) Å and *Cg*_2_···*Cg*_4_^iii^= 3.5988 (8) Å (symmetry codes (ii) = 2 - *x*, 1 - *y*, 1 - *z* and (iii) = 2 - *x*, 2 - *y*, 2 - *z*). *Cg*1, *Cg*2, *Cg*3, and *Cg*4 are the centroids of the N1–N2/C7–C8/C6–C17, C1–C6, C8–C12/C17, and C12–C17 rings, respectively.

### Experimental

The title compound was synthesized by adding a solution of 1,8-diaminonaphthalene (0.50 g, 3.16 mmol) in ethanol (20 ml) dropwise to a solution of *o*-vanillin (0.96 g, 6.32 mmol) in ethanol (10 ml). The reaction mixture was stirred for 0.5 h at room temperature and a pale-orange precipitate was obtained. After filtration, the pale-orange solid was washed with diethyl ether. A solution of the pale-orange solid (0.20 g, 0.47 mmol) in ethanol (20 ml) was slowly added to a solution of CoCl_2_.6H_2_O (0.11 g, 0.47 mmol) in 10 ml of ethanol followed by triethylamine (0.06 ml, 0.47 mmol). The mixture was refluxed for 3 h. The title compound was obtained as a purple solid and washed with diethyl ether. The purple needle-shaped single crystals of the unexpected perimidine derivative of the title compound suitable for *x*-ray structure determination were recrystallized from ethanol by slow evaporation of the solvent at room temperature over several days, Mp. 507–508 K.

### Refinement

All H atoms (except H13) were located in difference maps and refined isotropically. H13 was refined with *U*_iso_ constrained to be 1.2*U*_eq_ (C13). The highest residual electron density peak is located at 0.68 Å from C16 and the deepest hole is located at 0.48 Å from C16.

### Crystal data

**Table:**

C_18_H_12_N_2_O_3_	*F*(000) = 1264
*M**_r_* = 304.30	*D*_x_ = 1.493 Mg m^−^^3^
Monoclinic, *C*2/*c*	Melting point = 507–508 K
Hall symbol: -C 2yc	Mo *K*α radiation, λ = 0.71073 Å
*a* = 25.4718 (17) Å	Cell parameters from 6529 reflections
*b* = 7.0666 (3) Å	θ = 1.6–36.3°
*c* = 15.0815 (6) Å	µ = 0.10 mm^−^^1^
β = 94.373 (3)°	*T* = 100 K
*V* = 2706.8 (2) Å^3^	Needle, purple
*Z* = 8	0.67 × 0.11 × 0.05 mm

### Data collection

**Table:**

Bruker APEX DUO CCD area-detector diffractometer	6529 independent reflections
Radiation source: sealed tube	3471 reflections with *I* > 2σ(*I*)
graphite	*R*_int_ = 0.072
φ and ω scans	θ_max_ = 36.3°, θ_min_ = 1.6°
Absorption correction: multi-scan (*SADABS*; Bruker, 2005)	*h* = −42→34
*T*_min_ = 0.933, *T*_max_ = 0.994	*k* = −11→11
42939 measured reflections	*l* = −24→25

### Refinement

**Table:**

Refinement on *F*^2^	Primary atom site location: structure-invariant direct methods
Least-squares matrix: full	Secondary atom site location: difference Fourier map
*R*[*F*^2^ > 2σ(*F*^2^)] = 0.066	Hydrogen site location: inferred from neighbouring sites
*wR*(*F*^2^) = 0.210	All H-atom parameters refined
*S* = 1.02	*w* = 1/[σ^2^(*F*_o_^2^) + (0.110*P*)^2^] where *P* = (*F*_o_^2^ + 2*F*_c_^2^)/3
6529 reflections	(Δ/σ)_max_ = 0.001
255 parameters	Δρ_max_ = 0.57 e Å^−^^3^
0 restraints	Δρ_min_ = −0.29 e Å^−^^3^

### Special details

**Table:**

Experimental. The crystal was placed in the cold stream of an Oxford Cryosystems Cobra open-flow nitrogen cryostat (Cosier & Glazer, 1986) operating at 100.0 (1) K.
Geometry. All e.s.d.'s (except the e.s.d. in the dihedral angle between two l.s. planes) are estimated using the full covariance matrix. The cell e.s.d.'s are taken into account individually in the estimation of e.s.d.'s in distances, angles and torsion angles; correlations between e.s.d.'s in cell parameters are only used when they are defined by crystal symmetry. An approximate (isotropic) treatment of cell e.s.d.'s is used for estimating e.s.d.'s involving l.s. planes.
Refinement. Refinement of *F*^2^ against ALL reflections. The weighted *R*-factor *wR* and goodness of fit *S* are based on *F*^2^, conventional *R*-factors *R* are based on *F*, with *F* set to zero for negative *F*^2^. The threshold expression of *F*^2^ > σ(*F*^2^) is used only for calculating *R*-factors(gt) *etc*. and is not relevant to the choice of reflections for refinement. *R*-factors based on *F*^2^ are statistically about twice as large as those based on *F*, and *R*- factors based on ALL data will be even larger.

### Fractional atomic coordinates and isotropic or equivalent isotropic displacement parameters (Å^2^)

**Table:**

	*x*	*y*	*z*	*U*_iso_*/*U*_eq_	
O1	1.20986 (4)	0.86652 (17)	0.36853 (7)	0.0390 (3)	
O2	0.93604 (4)	0.76137 (15)	0.66009 (7)	0.0305 (2)	
H1O2	0.9692 (9)	0.784 (3)	0.6441 (15)	0.068 (7)*	
O3	0.83728 (4)	0.69274 (15)	0.67439 (7)	0.0307 (2)	
N1	1.00314 (4)	0.71962 (15)	0.40931 (7)	0.0230 (2)	
N2	1.01534 (4)	0.77988 (14)	0.56606 (7)	0.0217 (2)	
C1	0.92971 (5)	0.68964 (16)	0.50224 (9)	0.0213 (2)	
C2	0.90819 (5)	0.70778 (17)	0.58472 (9)	0.0229 (3)	
C3	0.85404 (5)	0.66982 (18)	0.59105 (9)	0.0245 (3)	
C4	0.82297 (5)	0.61425 (18)	0.51641 (10)	0.0269 (3)	
H4	0.7846 (8)	0.588 (3)	0.5190 (13)	0.053 (5)*	
C5	0.84470 (5)	0.59241 (19)	0.43503 (9)	0.0271 (3)	
H5	0.8242 (6)	0.542 (2)	0.3839 (11)	0.032 (4)*	
C6	0.89730 (5)	0.62922 (18)	0.42762 (9)	0.0242 (3)	
H6	0.9121 (6)	0.608 (3)	0.3716 (12)	0.039 (5)*	
C7	0.98576 (5)	0.73220 (16)	0.49261 (9)	0.0208 (2)	
C8	1.05351 (5)	0.75829 (17)	0.40160 (9)	0.0222 (2)	
C9	1.07449 (5)	0.7429 (2)	0.31468 (9)	0.0271 (3)	
H9	1.0495 (6)	0.696 (2)	0.2625 (11)	0.027 (4)*	
C10	1.12551 (5)	0.7780 (2)	0.30472 (9)	0.0281 (3)	
H10	1.1420 (6)	0.761 (2)	0.2446 (11)	0.031 (4)*	
C11	1.16293 (5)	0.83502 (19)	0.37912 (9)	0.0269 (3)	
C12	1.14203 (5)	0.84961 (17)	0.46741 (9)	0.0233 (3)	
C13	1.17405 (5)	0.89476 (19)	0.54295 (9)	0.0268 (3)	
H13	1.2155 (6)	0.916 (2)	0.5341 (11)	0.032*	
C14	1.15283 (5)	0.90293 (19)	0.62651 (10)	0.0284 (3)	
H14	1.1753 (6)	0.931 (2)	0.6784 (10)	0.029 (4)*	
C15	1.10056 (5)	0.86583 (18)	0.63559 (9)	0.0257 (3)	
H15	1.0859 (6)	0.865 (2)	0.6931 (12)	0.036 (4)*	
C16	1.06736 (5)	0.81935 (16)	0.55893 (9)	0.0218 (2)	
C17	1.08818 (5)	0.81121 (16)	0.47560 (8)	0.0206 (2)	
C18	0.78320 (6)	0.6497 (2)	0.68447 (12)	0.0338 (3)	
H18A	0.7621 (7)	0.737 (3)	0.6464 (12)	0.039 (5)*	
H18B	0.7746 (6)	0.521 (3)	0.6683 (10)	0.032 (4)*	
H18C	0.7805 (7)	0.666 (2)	0.7467 (12)	0.035 (4)*	

### Atomic displacement parameters (Å^2^)

**Table:**

	*U*^11^	*U*^22^	*U*^33^	*U*^12^	*U*^13^	*U*^23^
O1	0.0255 (5)	0.0525 (7)	0.0408 (6)	−0.0050 (5)	0.0157 (4)	0.0022 (5)
O2	0.0225 (5)	0.0393 (6)	0.0313 (5)	−0.0060 (4)	0.0121 (4)	−0.0095 (4)
O3	0.0201 (4)	0.0387 (6)	0.0353 (5)	−0.0036 (4)	0.0158 (4)	−0.0050 (4)
N1	0.0213 (5)	0.0227 (5)	0.0261 (6)	0.0006 (4)	0.0090 (4)	0.0043 (4)
N2	0.0189 (5)	0.0195 (5)	0.0278 (6)	−0.0018 (4)	0.0099 (4)	−0.0012 (4)
C1	0.0201 (5)	0.0168 (5)	0.0282 (6)	0.0001 (4)	0.0088 (5)	0.0031 (4)
C2	0.0204 (6)	0.0202 (5)	0.0290 (7)	−0.0007 (4)	0.0089 (5)	−0.0003 (4)
C3	0.0222 (6)	0.0220 (5)	0.0309 (7)	0.0002 (4)	0.0112 (5)	0.0011 (5)
C4	0.0186 (6)	0.0244 (6)	0.0383 (8)	−0.0017 (5)	0.0073 (5)	0.0059 (5)
C5	0.0235 (6)	0.0268 (6)	0.0313 (7)	−0.0019 (5)	0.0036 (5)	0.0066 (5)
C6	0.0245 (6)	0.0234 (6)	0.0254 (6)	−0.0002 (4)	0.0066 (5)	0.0054 (5)
C7	0.0213 (5)	0.0171 (5)	0.0253 (6)	0.0013 (4)	0.0092 (5)	0.0019 (4)
C8	0.0212 (5)	0.0196 (5)	0.0266 (6)	0.0013 (4)	0.0079 (5)	0.0041 (4)
C9	0.0265 (6)	0.0316 (6)	0.0244 (6)	0.0006 (5)	0.0096 (5)	0.0042 (5)
C10	0.0273 (6)	0.0330 (7)	0.0254 (7)	0.0006 (5)	0.0118 (5)	0.0051 (5)
C11	0.0249 (6)	0.0271 (6)	0.0304 (7)	0.0000 (5)	0.0127 (5)	0.0043 (5)
C12	0.0204 (5)	0.0213 (5)	0.0297 (6)	−0.0008 (4)	0.0109 (5)	0.0026 (5)
C13	0.0219 (6)	0.0261 (6)	0.0336 (7)	−0.0046 (5)	0.0090 (5)	0.0000 (5)
C14	0.0248 (6)	0.0295 (6)	0.0319 (7)	−0.0057 (5)	0.0077 (5)	−0.0041 (5)
C15	0.0245 (6)	0.0263 (6)	0.0273 (7)	−0.0051 (5)	0.0100 (5)	−0.0033 (5)
C16	0.0211 (5)	0.0175 (5)	0.0281 (6)	−0.0020 (4)	0.0104 (4)	−0.0016 (4)
C17	0.0210 (5)	0.0168 (5)	0.0253 (6)	0.0000 (4)	0.0094 (4)	0.0024 (4)
C18	0.0192 (6)	0.0437 (9)	0.0405 (9)	−0.0047 (6)	0.0145 (6)	−0.0017 (7)

### Geometric parameters (Å, °)

**Table:**

O1—C11	1.2384 (16)	C8—C17	1.4196 (18)
O2—C2	1.3474 (16)	C8—C9	1.4568 (19)
O2—H1O2	0.91 (2)	C9—C10	1.3427 (19)
O3—C3	1.3676 (16)	C9—H9	1.028 (16)
O3—C18	1.4301 (16)	C10—C11	1.472 (2)
N1—C8	1.3256 (16)	C10—H10	1.035 (17)
N1—C7	1.3663 (17)	C11—C12	1.4747 (18)
N2—C7	1.3345 (17)	C12—C13	1.3868 (19)
N2—C16	1.3664 (16)	C12—C17	1.4127 (17)
C1—C2	1.4030 (18)	C13—C14	1.4098 (19)
C1—C6	1.4102 (19)	C13—H13	1.085 (16)
C1—C7	1.4769 (17)	C14—C15	1.3739 (18)
C2—C3	1.4155 (17)	C14—H14	0.955 (15)
C3—C4	1.3827 (19)	C15—C16	1.4179 (18)
C4—C5	1.393 (2)	C15—H15	0.970 (17)
C4—H4	1.000 (19)	C16—C17	1.4019 (18)
C5—C6	1.3777 (18)	C18—H18A	0.976 (18)
C5—H5	0.966 (16)	C18—H18B	0.963 (17)
C6—H6	0.963 (18)	C18—H18C	0.954 (18)
			
C2—O2—H1O2	105.4 (14)	C9—C10—C11	122.77 (12)
C3—O3—C18	116.32 (11)	C9—C10—H10	122.6 (9)
C8—N1—C7	116.80 (11)	C11—C10—H10	114.6 (9)
C7—N2—C16	118.40 (11)	O1—C11—C10	121.75 (12)
C2—C1—C6	119.38 (11)	O1—C11—C12	121.49 (13)
C2—C1—C7	120.97 (11)	C10—C11—C12	116.75 (11)
C6—C1—C7	119.65 (11)	C13—C12—C17	119.11 (12)
O2—C2—C1	123.88 (11)	C13—C12—C11	121.84 (12)
O2—C2—C3	116.70 (11)	C17—C12—C11	119.03 (12)
C1—C2—C3	119.41 (12)	C12—C13—C14	120.14 (12)
O3—C3—C4	125.62 (12)	C12—C13—H13	116.6 (8)
O3—C3—C2	114.43 (12)	C14—C13—H13	123.2 (8)
C4—C3—C2	119.95 (12)	C15—C14—C13	121.45 (13)
C3—C4—C5	120.50 (12)	C15—C14—H14	118.9 (9)
C3—C4—H4	121.5 (11)	C13—C14—H14	119.6 (9)
C5—C4—H4	118.0 (11)	C14—C15—C16	119.02 (12)
C6—C5—C4	120.32 (13)	C14—C15—H15	122.2 (10)
C6—C5—H5	118.3 (9)	C16—C15—H15	118.7 (10)
C4—C5—H5	121.2 (9)	N2—C16—C17	119.84 (11)
C5—C6—C1	120.42 (12)	N2—C16—C15	120.32 (11)
C5—C6—H6	119.3 (10)	C17—C16—C15	119.84 (11)
C1—C6—H6	120.2 (10)	C16—C17—C12	120.44 (12)
N2—C7—N1	125.29 (11)	C16—C17—C8	117.41 (11)
N2—C7—C1	117.29 (11)	C12—C17—C8	122.12 (11)
N1—C7—C1	117.42 (11)	O3—C18—H18A	107.1 (10)
N1—C8—C17	122.25 (12)	O3—C18—H18B	112.2 (9)
N1—C8—C9	119.17 (12)	H18A—C18—H18B	110.1 (14)
C17—C8—C9	118.57 (11)	O3—C18—H18C	102.7 (10)
C10—C9—C8	120.76 (13)	H18A—C18—H18C	115.0 (15)
C10—C9—H9	121.4 (9)	H18B—C18—H18C	109.5 (14)
C8—C9—H9	117.7 (9)		
			
C6—C1—C2—O2	178.87 (11)	C8—C9—C10—C11	−0.6 (2)
C7—C1—C2—O2	−0.90 (19)	C9—C10—C11—O1	179.92 (13)
C6—C1—C2—C3	−1.62 (18)	C9—C10—C11—C12	1.0 (2)
C7—C1—C2—C3	178.60 (10)	O1—C11—C12—C13	−1.6 (2)
C18—O3—C3—C4	1.88 (19)	C10—C11—C12—C13	177.40 (12)
C18—O3—C3—C2	−177.96 (12)	O1—C11—C12—C17	−179.71 (12)
O2—C2—C3—O3	−0.19 (17)	C10—C11—C12—C17	−0.76 (17)
C1—C2—C3—O3	−179.73 (10)	C17—C12—C13—C14	−0.32 (19)
O2—C2—C3—C4	179.96 (11)	C11—C12—C13—C14	−178.48 (12)
C1—C2—C3—C4	0.42 (18)	C12—C13—C14—C15	0.4 (2)
O3—C3—C4—C5	−178.86 (12)	C13—C14—C15—C16	−0.3 (2)
C2—C3—C4—C5	0.98 (19)	C7—N2—C16—C17	0.73 (17)
C3—C4—C5—C6	−1.1 (2)	C7—N2—C16—C15	−178.36 (11)
C4—C5—C6—C1	−0.1 (2)	C14—C15—C16—N2	179.33 (12)
C2—C1—C6—C5	1.47 (18)	C14—C15—C16—C17	0.24 (19)
C7—C1—C6—C5	−178.75 (11)	N2—C16—C17—C12	−179.29 (10)
C16—N2—C7—N1	−0.21 (18)	C15—C16—C17—C12	−0.20 (18)
C16—N2—C7—C1	179.80 (10)	N2—C16—C17—C8	−0.99 (17)
C8—N1—C7—N2	−0.03 (18)	C15—C16—C17—C8	178.10 (11)
C8—N1—C7—C1	179.96 (10)	C13—C12—C17—C16	0.24 (18)
C2—C1—C7—N2	2.97 (17)	C11—C12—C17—C16	178.45 (10)
C6—C1—C7—N2	−176.80 (11)	C13—C12—C17—C8	−177.98 (11)
C2—C1—C7—N1	−177.02 (10)	C11—C12—C17—C8	0.23 (18)
C6—C1—C7—N1	3.21 (16)	N1—C8—C17—C16	0.77 (17)
C7—N1—C8—C17	−0.26 (17)	C9—C8—C17—C16	−178.10 (11)
C7—N1—C8—C9	178.60 (11)	N1—C8—C17—C12	179.04 (11)
N1—C8—C9—C10	−178.90 (12)	C9—C8—C17—C12	0.17 (17)
C17—C8—C9—C10	0.01 (19)		

### Hydrogen-bond geometry (Å, °)

**Table:**

*D*—H···*A*	*D*—H	H···*A*	*D*···*A*	*D*—H···*A*
O2—H1O2···N2	0.91 (2)	1.73 (2)	2.5583 (15)	151 (2)
C15—H15···O2^i^	0.970 (18)	2.437 (18)	3.3686 (17)	160.8 (12)

Symmetry codes:  (i) −*x*+2, *y*, −*z*+3/2.
